# Concomitant percutaneous coronary intervention and transcatheter aortic valve replacement for aortic stenosis complicated with acute STEMI: a case report and literature review

**DOI:** 10.3389/fcvm.2023.1291089

**Published:** 2023-11-28

**Authors:** Chengyi Xu, Hanhua Hu, Xi Su

**Affiliations:** Department of Cardiology, Wuhan Asia Heart Hospital, Wuhan, China

**Keywords:** percutaneous coronary intervention, transcatheter aortic valve replacement, aortic stenosis, acute ST-segment elevation myocardial infarction, cardiogenic shock, case report

## Abstract

Aortic stenosis (AS) complicated with acute ST-segment elevation myocardial infarction (STEMI) is a life-threatening emergency with high mortality. A 75-year-old male patient attended the emergency department of Wuhan Asia Heart Hospital in December 2021 with chest pain for 2 days and exacerbation for 1 h. The electrocardiogram (ECG) indicated atrial fibrillation with rapid ventricular response and ST-segment depression. Echocardiography showed severe AS and mild/moderate aortic insufficiency. The patient refused coronary angiography and further invasive procedures and then requested discharge, but he had recurrent chest pain on the third day. The ECG showed an extensive anterior wall STEMI. During preoperative preparation, he suffered from cardiogenic shock (CS). Concomitant percutaneous coronary intervention (PCI) and transcatheter aortic valve replacement (TAVR) was performed, but he died of CS and multiple organ failure 4 days after surgery. Patients with AS and STEMI might be susceptible to CS during perioperative period of concomitant PCI and TAVR, which requires proactive prevention.

## Introduction

Aortic stenosis (AS) is a common heart valve disease with poor prognosis for symptomatic patients, particularly the elderly (1). Acute ST-segment elevation myocardial infarction (STEMI) is the most critical clinical type of coronary artery disease (CAD), which can lead to severe myocardial damage and even cardiogenic shock (CS) (2). AS complicated with STEMI is a life-threatening emergency with high mortality (3). Previous study has indicated the feasibility and safety of combined percutaneous coronary intervention (PCI) in high-risk patients with severe AS undergoing transcatheter aortic valve replacement (TAVR) (4). However, concomitant PCI and TAVR has been rarely reported for AS complicated with STEMI and CS. Therefore, this study reports a patient with severe AS suffering from acute STEMI and CS who was treated by concomitant PCI and TAVR.

## Case presentation

A 75-year-old male patient attended the emergency department of Wuhan Asia Heart Hospital in December 2021 with complaints of chest pain for 2 days and exacerbation for 1 h. The patient had hypertension and hyperlipidemia for 4 years and smoked for more than 50 years. Physical examination showed absolute arrhythmia, systolic ejection murmurs in the first and second auscultation areas of aortic valve, and mild sight-like murmurs during diastole. Vascular murmurs could be found in the bilateral neck and femoral artery. Hyper-sensitive cardiac troponin I (hs-cTnI) was 0.350 ng/ml (reference: 0–0.03 ng/ml) and N-terminal pro-brain natriuretic peptide (NT-proBNP) was 3,008.4 pg/ml (reference: 0–125 pg/ml). Echocardiography (ECG) indicated atrial fibrillation (AF) with rapid ventricular response and ST-segment depression. Transthoracic echocardiography (TTE) showed severe AS, mild/moderate aortic insufficiency (AI), and left ventricular ejection fraction of 52%. He was preliminarily diagnosed as severe AS with mild-to-moderate AI accompanied by acute non-ST-segment elevation myocardial infarction (NSTEMI), Killip II, with New York Heart Association (NYHA) class III. He refused emergency coronary angiography (CAG) and computer tomography angiography (CTA) and requested discharge on the next day, but he suffered from recurrent chest pain for 1 h on the third day. The ECG indicated STEMI in extensive anterior-wall, and hs-cTnI was 3.105 ng/ml. He was diagnosed with severe AS complicated with acute STEMI; one-stop PCI combined with TAVR was performed. During preoperative preparation, the patient had dyspnea with orthopnea, and blood pressure decreased to 80/50 mmHg, accompanied by oliguria and peripheral dampness and cold. CS was suspected, and dopamine and norepinephrine were administered. Emergency endotracheal intubation and invasive ventilation were performed.

**Figure 1:**
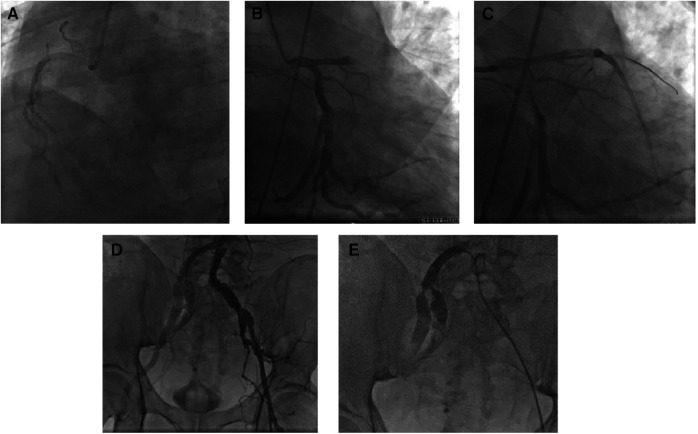
Coronary artery intervention treatment. The emergency coronary angiography results showed a left-dominant coronary artery, no severe stenosis in the right coronary artery (**A**), and no significant stenosis in the circumflex branch (**B**), but acute complete occlusion of the proximal segment of the left anterior descending branch (**C**). Angiography of the abdominal aorta-iliac artery bifurcation revealed diffuse narrowing of the left femoral artery (intravascular diameter <5 mm) and multiple ulcers and aneurysmal dilatation of the external iliac artery (**D**); selective angiography of the right iliac artery showed focal stenosis at the bifurcation of the right external-internal iliac artery (**E**).

**Figure 2:**
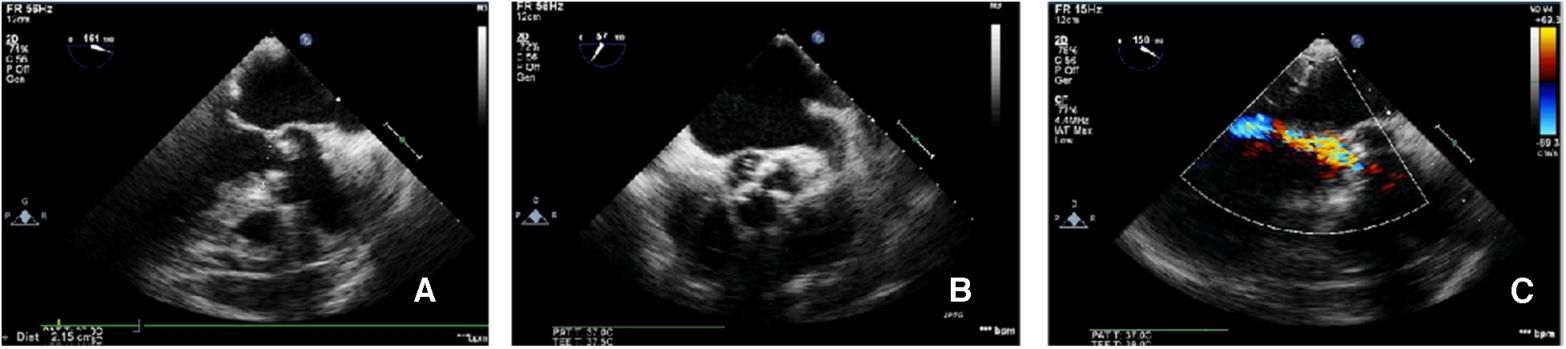
TEE-measured virtual valve annulus diameter. The TEE-measured virtual aortic valve annulus diameter was 21.5 mm (**A**); the tricuspid aortic valve leaflets were severely calcified (**B**); color Doppler ultrasound showed mild-to-moderate aortic valve regurgitation (**C**).

**Figure 3:**
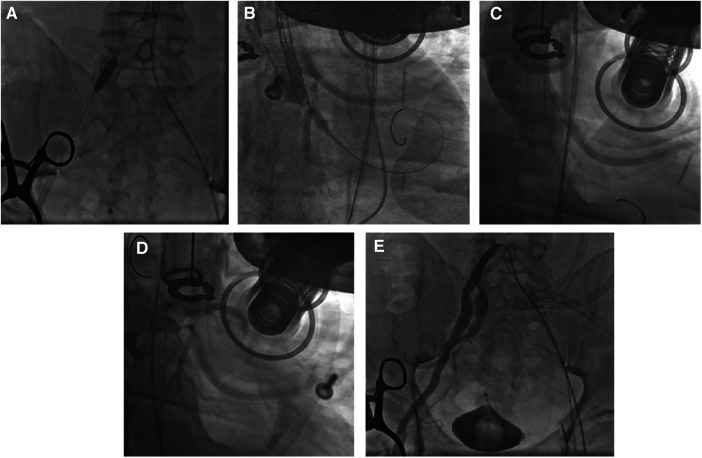
TAVR process. After the 4.0 mm × 20 mm peripheral balloon was sent to the stenosis site of the external-internal iliac artery bifurcation through a 20Fr hydrophilic-coated guiding sheath, balloon angioplasty was performed with 6 atm to dilate the lesion vessel (**A**). The valve was quickly delivered to the aortic root through the catheter under mechanical chest compressions and was released after proper positioning (**B**). Aortic angiography showed no paravalvular leaks (**C**). Selective left coronary angiography indicated good coronary artery visualization (**D**). Selective right iliac artery angiography confirmed residual stenosis of less than 30% after balloon angioplasty of the stenosis site of the external-internal iliac artery bifurcation, with no local vascular complications such as dissection (**E**).

CAG was performed via the left femoral artery route (Figures 1A,B). The anterior descending artery was the culprit vessel with proximal acute occlusion, and a 3.5 mm × 23 mm drug-eluting stent was implanted (Figure 1C). A temporary pacemaker electrode was implanted at the apex of right ventricle. The angiography showed diffuse stenosis of the left common femoral artery, multiple ulcers and tumor-like dilatation of the external iliac artery, and limited stenosis of the right external iliac-internal iliac artery with a degree of 70% (Figures 1D,E). Transesophageal echocardiography (TEE) indicated that the inner diameter of the aortic valve annulus was 21.5 mm (Figure 2). Due to vascular stenosis, the femoral artery route was not suitable for TAVR. The feasibility of the carotid artery route was considered. However, the patient had a sudden circulatory collapse. Continuous chest compressions were performed and emergency incision exposed the right femoral artery. A peripheral balloon was delivered to the external-internal iliac artery bifurcation through a hydrophilic-coated guiding sheath, and lesional balloon angioplasty was performed (Figure 3A). Then, under continuous mechanical compression, a valve system was rapidly delivered to the aortic root, and chest compressions were then stopped. No paravalvular leakage was found using ascending aortography (Figures 3B,C). The selective left CAG showed a well-developed coronary artery (Figure 3D). Angiography of the right iliac artery showed that residual stenosis of the external iliac artery to the internal iliac artery bifurcation was less than 30%, and no local vascular complications such as dissection were found (Figure 3E). Thus, he was treated with veno-arterial extracorporeal membrane oxygenation (VA-ECMO) through the right femoral vein and artery, and intra-aortic balloon pump (IABP) through the left femoral artery.

After resuscitation, his autonomic circulation was rescued with ECMO combined with IABP-assisted circulation, and the temporary pacemaker was retained. However, he died of CS combined with multiple organ failure (MOF) 4 days after the surgery.

## Discussion

This study reports a patient with severe AS suffering from acute STEMI and CS. He was treated by concomitant PCI and TAVR but died of CS and MOF 4 days after the surgery. Patients with AS and STEMI might be susceptible to CS during the perioperative period of concomitant PCI and TAVR, which requires proactive prevention.

PCI combined with TAVR surgery could be divided into preoperative PCI, intraoperative one-stop PCI, and postoperative PCI for TAVR surgery (5). For AS patients with NSTEMI, the choice of simultaneous or elective PCI combined with TAVR depends on various factors, including old age, operation time, contrast agent usage, organ function, and complexity of CAD (6). Simple coronary artery lesions can be treated with simultaneous PCI and TAVR, whereas complex lesions may require PCI performed 10 days prior to TAVR to improve patient tolerance to ischemia during TAVR (7). Elective operations should be considered for elderly patients and those with organ dysfunction who cannot tolerate long operation times and high-dose contrast agents. The review by Perez et al. systematically elucidates the timing and advantages and disadvantages of coronary intervention therapy for patients with aortic valve disease (6). We can assess the risk of coronary ostia occlusion in the left coronal tangent position during balloon sizing. Alternatively, the height of the coronary ostia from the valve annulus can be measured using three-dimensional transesophageal echocardiography. There are two predominant stenting techniques described in the literature for the patients who are at risk of coronary occlusion. The first is the more traditional ostial stenting technique, in which the stent is deployed just protruding into the aorta, but this remains unproven in TAVR (8). The second is the snorkel stenting technique, in which the stent is implanted in the ostia then snorkeled up alongside the TAVR valve into the aorta (9).

For patients with severe AS and STEMI, it remains controversial whether PCI should be performed concurrently or electively with TAVR. In this case, an elderly male with complete occlusion of the proximal left anterior descending branch required PCI. He had high gradient AS, and aortic valve replacement was recommended (10). Surgical aortic valve replacement (SAVR) was not suitable due to his peripheral vascular condition and high risk (11). Thus, one-stop PCI with TAVR was performed. Due to the possibility of prolonged operation time, implanting a circulatory support device during or before the procedure was necessary.

Konami et al. reported an 84-year-old patient who was treated with primary PCI for STEMI and experienced CS after surgery and was treated with TAVR and insertion of an Impella CP device (12). Three months after admission, the patient was in good condition at his outpatient follow-up visit, with a New York Heart Association functional class II. Transthoracic echocardiography showed improved cardiac function. El Tahlawy et al. reported a 57-year-old patient who was treated initially with PCI supported by IABP, but experienced CS after PCI (13) (Table 1). A combination of early ECMO followed by PABV and TAVR was performed for critical bicuspid AS. However, one-stop PCI with TAVR has been rarely reported for AS complicated with STEMI and CS. IABP could increase cardiac output by reducing afterload and improving myocardial oxygen perfusion (14). It has been reported that reported that patients with severe AS successfully underwent PCI with TAVR under the support of IABP (15). A left ventricular assist device (Impella) provides a larger cardiac volume than IABP, reduces left ventricular end-diastolic, increases mean arterial pressure, and decreases myocardial oxygen consumption (16). However, it is contraindicated in patients with severe AS with a valve area less than 0.6 cm^2^ (17). Thus, IABP was more appropriate for this patient. ECMO can adequately maintain patient oxygenation and increase mean arterial pressure and cardiac index (18). Thus, ECMO combined with IABP-assisted circulation was used to rescue the autonomic circulation of this case. In patients with acute myocardial infarction complicated with CS, VA-ECMO combined with IABP instrumental circulatory support significantly reduced mortality compared with VA-ECMO alone (19). For STEMI patients with CS undergoing PCI, the application of Impella or VA-ECMO before PCI significantly reduced mortality, whereas the use of IABP before and after PPCI had no effect on mortality (20). Huang et al. reported that for STEMI patients, ECMO implantation prior to PCI improved survival at six months and improved short- and long-term outcomes (21). Radsel et al*.* suggested that the survival rate of patients with ECMO before TAVR was higher than that those with ECMO after CS during TAVR (22). In this patient, the failure to implant ECMO prior to PCI and the limited use of Impella might have been associated with the unsatisfactory outcome. The patient died of CS and MOF 4 days after operation. This case presented surgical difficulties due to the patient's critical condition, including old age, complete occlusion of the left anterior descending branch, and poor peripheral vascular conditions with bilateral iliac artery aneurysms and stenosis. Patients with high-risk PCI combined with severe AS are at high risk for CS. The preoperative implantation percutaneous mechanical circulation auxiliary device might be beneficial to the prognosis of patients.

**Table 1:** A review of the diagnosis and treatment of patients with acute STEMI and severe AS.

Author	Publication time	Sex	Age, years	Diagnosis	Treatment
Konami et al. (9)	February, 2021	Male	84	Acute STEMI and severe AS	Primary PCI; he experienced CS a few hours later and was treated with TAVR and the insertion of an Impella CP device.
El Tahlawy et al. (10)	March, 2022	Male	57	Acute posterior STEMI and severe AS	Initially PCI supported by IABP; the procedure was successful, but he experienced CS after PCI; a combination of early ECMO followed by PABV and TAVR was performed for critical bicuspid AS.

In conclusion, this study highlighted that patients with AS and STEMI might be susceptible to CS during the perioperative period of concomitant PCI and TAVR, which requires proactive prevention.

## Data Availability

The original contributions presented in the study are included in the article/Supplementary Material, further inquiries can be directed to the corresponding author.
